# UHGAN: a dual-phase GAN with Hough-transform constraints for accurate farmland road extraction

**DOI:** 10.3389/fnbot.2025.1691300

**Published:** 2025-10-13

**Authors:** Xinliang Wang, Yuan Ma

**Affiliations:** ^1^Nanxun Innovation Institute, Zhejiang University of Water Resources and Electric Power, Hangzhou, China; ^2^Huzhou University, HuZhou, China

**Keywords:** generative adversarial network, Hough transform, breakpoint repair, farmland road extraction, semantic segmentation

## Abstract

**Introduction:**

Traditional methods for farmland road extraction, such as U-Net, often struggle with complex noise and geometric features, leading to discontinuous extraction and insufficient sensitivity. To address these limitations, this study proposes a novel dual-phase generative adversarial network (GAN) named UHGAN, which integrates Hough-transform constraints.

**Methods:**

We designed a cascaded U-Net generator within a two-stage GAN framework. The Stage 1 GAN combines a differentiable Hough transform loss with cross-entropy loss to generate initial road masks. Subsequently, the Stage 2 U-Net refines these masks by repairing breakpoints and suppressing isolated noise.

**Results:**

When evaluated on the WHU RuR+rural road dataset, the proposed UHGAN method achieved an accuracy of 0.826, a recall of 0.750, and an F1-score of 0.789. This represents a significant improvement over the single-stage U-Net (F1 = 0.756) and ResNet (F1 = 0.762) baselines.

**Discussion:**

The results demonstrate that our approach effectively mitigates the issues of discontinuous extraction caused by the complex geometric shapes and partial occlusion characteristic of farmland roads. The integration of Hough-transform loss, an technique that has received limited attention in prior studies, proves to be highly beneficial. This method shows considerable promise for practical applications in rural infrastructure planning and precision agriculture.

## 1 Introduction

High-standard farmland is characterized by its concentration, contiguity, and supporting infrastructure, including field roads that are essential for agricultural production and management. The accurate extraction of road networks from such areas is critical for enhancing construction planning and operational efficiency. However, existing methods, primarily based on U-Net (Ronneberger et al., 2015) and similar architectures, often struggle with complex rural scenes where occlusions from buildings, waterways, and vegetation complicate road connectivity and geometry.

Generally, deep learning models can be categorized into discriminative models and generative models. Discriminative models have advanced rapidly due to innovations such as backpropagation (BP) (Rumelhart et al., 1986) and Dropout (Zhang and Xu, 2024), In contrast, progress in generative models has been slower owing to challenges in model formulation and loss function definition. The development of this field gained momentum only after the introduction of a novel generative framework: Generative Adversarial Networks (GANs) (Goodfellow et al., 2020).

A GAN is a deep learning methodology comprising at least two modules—a generator and a discriminator—that enhance their respective output quality and predictive capability through adversarial training. This approach represents one of the most promising methods for unsupervised learning on complex distributions. The primary challenge in GANs lies in training dynamics. Since GANs require simultaneous optimization of two modules, identifying compatible parameters and methodologies that enable joint convergence is critical. Effective training strategies can significantly reduce training difficulty, accelerate convergence, and improve final accuracy, whereas suboptimal approaches may result in mode collapse or complete training failure. Prior research has demonstrated GAN applications in image generation domains such as artistic painting (Wang et al., 2023). Road generation fundamentally constitutes an image generation task. Consequently, GANs also exhibit strong potential for road generation applications. Notably, Generative Adversarial Networks (GANs) have shown unique advantages in capturing intricate data distributions and recovering detailed structures, making them suitable for road extraction from noisy remote sensing imagery. Furthermore, incorporating feature detection techniques such as the Hough transform can enhance the model’s ability to capture geometric regularity and linear continuity inherent in road networks.

Hough transform (Romanengo et al., 2022) is an image feature detection method based on parameter-space voting mechanism. It was initially developed to detect straight lines in images, and later expanded to recognize arbitrary shapes such as circles and ellipses. Its core idea is to map geometric features from image space to parameter space and determine target parameters through voting.

In remote sensing and street view images, roads typically manifest as linear or curved structures exhibiting characteristics such as continuity and directionality. The Hough transform adapts effectively to these shapes by: extracting directional features, detecting roads with varying widths and curvatures, and integrating edge points to reconstruct complete road profiles. Traditional road extraction models, such as U-Net, employ Cross Entropy Loss (Mao et al., 2023) or Dice Loss (Milletari et al., 2016). These focus on pixel-level classification accuracy but struggle to constrain geometric rationality in output results. Integrating Hough transform into the loss function provides explicit constraints to guide model learning of global road structural features.

In order to improve the extraction accuracy, generalization, and robustness of field roads, this paper is inspired by PLGAN (Abdelfattah et al., 2023) and designs a multi-layer joint learning method UHGAN based on U-Net. Unlike standard U-Net or existing GAN hybrids, UHGAN employs a cascaded learning strategy: the Stage 1 generator produces an initial road mask using a hybrid objective combining cross-entropy and Hough transform losses, emphasizing global geometric structure; the Stage 2 generator performs local refinement, repairing discontinuities and suppressing irrelevant noise, which helps U-Net network to further explore the geometric and directional features of roads and carry out fast extraction of high standard field roads.

The main contributions are as follows:

We propose UHGAN, a dual-stage GAN framework based on U-Net, which improves road extraction accuracy through adversarial training and multi-stage refinement.

We design a joint learning strategy where the Stage 1 generator captures coarse road geometry and the Stage 2 generator enhances connectivity and eliminates artifacts.

We introduce a Hough transform-based loss function to explicitly enforce geometric constraints, enabling more accurate detection of linear and curvilinear road features in high-noise environments.

## 2 Preparation

### 2.1 Theoretical framework of generative adversarial networks (GANs)

Generative Adversarial Networks (GANs) were proposed by Goodfellow et al. in 2014. Their core idea achieves data distribution modeling through adversarial competition between generators and discriminators (Goodfellow et al., 2014). This section systematically elaborates the fundamental theory and improvement methods of GANs.

The goal of generator G is to generate samples G (z) G (z) from the latent space z ∼ pz (z) that are similar to the true data distribution PDATA (x) PDATA (x), while discriminator DD needs to distinguish between the true data xx and the generated data G (z) G (z). The optimization objective of the original GAN is to minimize the adversarial loss between the generator and discriminator (Equation 1).


(1) $$\begin{array}{c} \min_{G}\max_{D}V(D,G)=E_{x∼p_{\text{data}}(x)}[\log D(x)]\\ +E_{z∼p_{z}(z)}[\log(1-D(G(z)))] \end{array}$$

However, the original GAN suffers from issues such as vanishing gradients and mode collapse. Researchers have subsequently proposed improvement solutions. For example, DCGAN introduces convolutional networks by replacing fully connected layers with strided convolutions, incorporates batch normalization (BatchNorm) for stable training, and adopts conditional generative adversarial networks that guide the generation process through conditional inputs. This expands GAN applications in controllable generation tasks. The GAN implemented in this study utilizes original remote sensing images and corresponding labels as conditional inputs for image generation.

GANs have demonstrated exceptional performance in domains including image generation (Liu et al., 2022) and image restoration (Huang et al., 2023), Nevertheless, their training process exhibits sensitivity to hyperparameters and may produce artifacts like blurred details and texture distortions in complex scenes, requiring further improvement (Guo et al., 2022). Nevertheless, GANs’ ability to model high-frequency details and contextual relationships makes them highly suitable for recovering geometrically consistent road networks under noisy conditions.

### 2.2 The structural characteristics and development of U-net network

U-Net was proposed by Ronneberger et al. in 2015 and was initially applied to medical image segmentation tasks. Its distinctive encoder-decoder architecture with skip connections has established it as the mainstream framework for pixel-level prediction tasks. The classic structure comprises three components: encoders, decoders, and skip connections. The encoder extracts multi-scale features through layered convolutions and downsampling, compressing spatial dimensions. Simultaneously, the decoder restores spatial resolution via transposed convolution or upsampling, fusing feature maps from corresponding encoder layers through skip connections to mitigate gradient vanishing. These connections integrate shallow and deep information, significantly enhancing pixel-level localization accuracy.

U-Net demonstrates strong performance in applications including medical image segmentation (Isensee et al., 2021) and remote sensing image processing (Cui et al., 2023). However, its substantial parameter count may incur high computational costs, and edge localization for low-contrast targets remains challenging. In particular, U-Net alone may struggle to maintain road connectivity under heavy occlusion or complex background noise. Integrating U-Net with adversarial training and geometric constraints—as proposed in our UHGAN framework—helps overcome these limitations by enhancing structural coherence and suppressing false positives.

### 2.3 The motivation and method of combining U-net and GAN

The combination of U-net’s pixel-level generation capability and GAN’s adversarial training mechanism provides a novel approach for image-to-image generation tasks: U-Net serves as the generator, with skip connections transmitting local details to the decoder, addressing the detail loss limitation in traditional CNN generators like DCGAN. The encoder-decoder structure simultaneously models global semantics and local textures, making it suitable for image restoration and super-resolution tasks (Han et al., 2022). Using the Pix2Pix framework (Tahmid et al., 2023), t the U-Net generator combines with a PatchGAN discriminator to learn image mapping relationships through paired data. For scenarios lacking paired data, introducing cycle consistency loss (Zhu et al., 2017) enables cross-domain image generation when integrated with U-net.

## 3 Introduction to UHGAN model

In this section, we will introduce the UHGAN. We will first provide an overview of our proposed method, and then introduce joint learning, GAN neural networks, and Hough transform loss in the following summary.

### 3.1 Overview

In order to enhance the model’s road extraction capability and reduce accuracy degradation from discontinuities or isolated points, we designed a two-stage joint training framework. While individual components such as GANs, PatchGAN, and cascaded U-Net have been explored in prior work, the novelty of UHGAN lies not merely in their combination, but in the structured and goal-oriented integration of a differentiable Hough transform loss within a dual-phase generative adversarial framework, explicitly designed to address the geometric and topological challenges of farmland road extraction. Unlike PLGAN (Abdelfattah et al., 2023) and Pix2Pix (Tahmid et al., 2023) which use geometric priors only in post-processing or as non-differentiable regularizers, UHGAN integrates a differentiable Hough transform loss directly into the adversarial training process. This allows the model to directly learn globally consistent road structures during adversarial training. Furthermore, the proposed two-stage refinement strategy is functionally specialized: the first stage focuses on recovering geometrically plausible road layouts under strong structural constraints, while the second stage acts as a connectivity-enhancing repair network that is trained purely under reconstruction loss to eliminate fractures and noise without compromising semantic consistency. This targeted division of structural generation and topological refinement—guided by a purpose-built hybrid loss system—has not been previously established in rural road extraction tasks, and represents a novel architectural paradigm that effectively balances pixel accuracy, geometric regularity, and connectivity integrity. First, we employ the U-Net network as the base architecture for our Stage 1 generator for preliminary training. Original remote sensing images and their corresponding labels serve as inputs to produce an initial prediction. Subsequently, we use the original labels combined with this Stage 1 prediction as new inputs, feeding them through a second U-Net generator to output the final prediction. Stages are trained jointly each iteration, gradients flow only within each stage (Stage 1 via adversarial + geometric losses; Stage 2 via L1), as Stage 1 outputs are detached.

**Figure 1:**
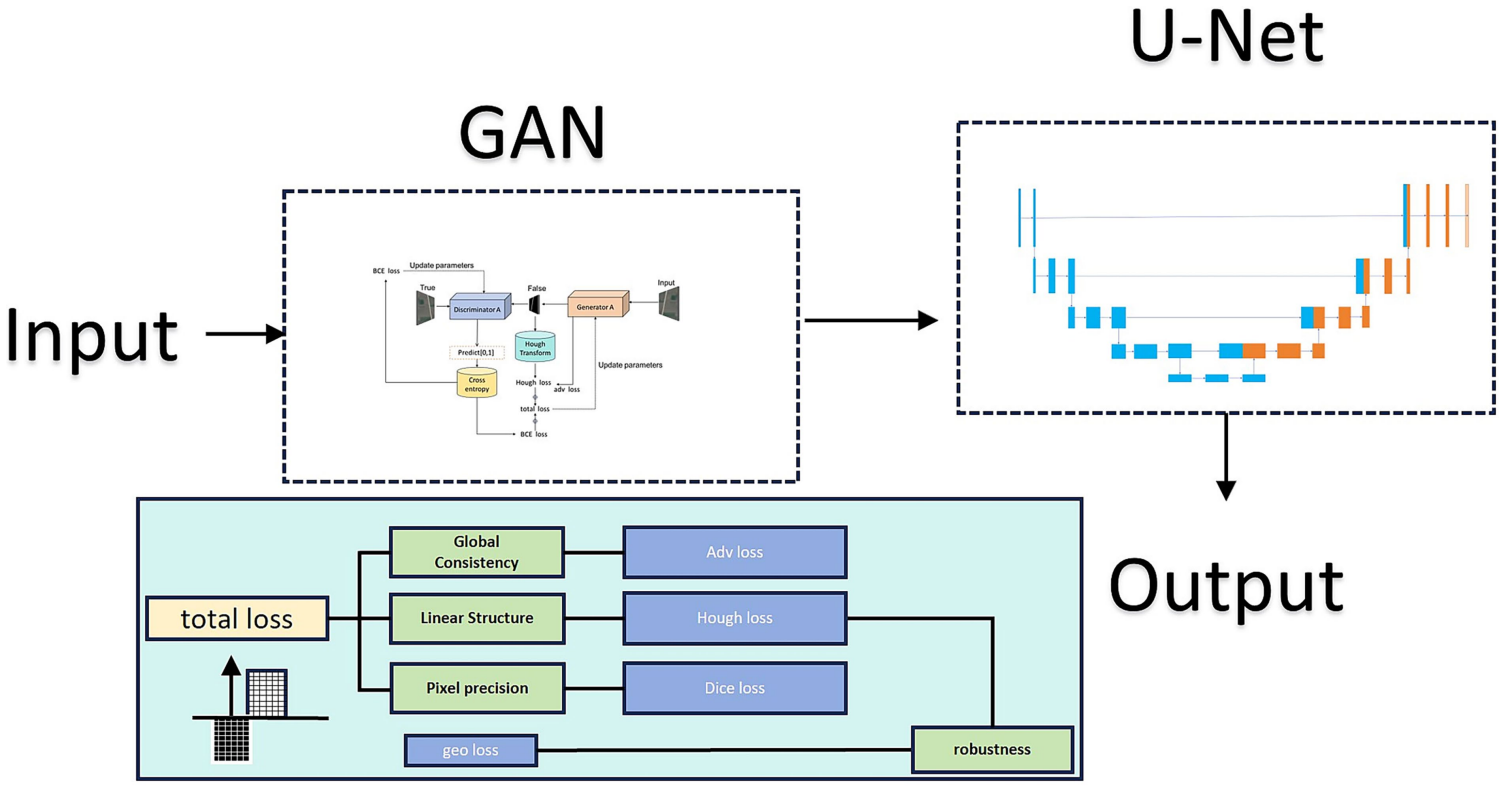
Architecture of the proposed UHGAN framework compared with a standard U-Net. UHGAN adopts a dual-stage design: Stage 1 employs a GAN generator with adversarial, segmentation, Hough-transform, and geometric consistency losses to capture global road structures, while Stage 2 uses a refinement U-Net to repair discontinuities and suppress noise. This joint training strategy ensures both spatial structural consistency and high prediction accuracy.

During Stage 1 GAN training, we implemented a U-net-based generator and incorporated Hough transform loss alongside pixel accuracy loss and adversarial loss. This multi-objective optimization scheme simultaneously addresses multiple aspects of road extraction, yielding results that better approximate real road visual characteristics. However, GAN limitations prevent complete detection and reconstruction of missing or isolated points. Therefore, we introduced a second U-Net layer to teach the model both road structure determination and isolated point removal. The model architecture is shown in Figure 1:

### 3.2 Multi level loss function combining Hough transform characteristics

Hough transform is a classical feature detection method, first proposed by Paul Hough in 1962 and later refined to its modern form by Duda and Hart (1972). It effectively detects parameterized shapes in images, particularly lines, circles, and ellipses. In road extraction tasks, Hough transform proves especially valuable since road networks typically exhibit structures with distinct linear characteristics. This technique transforms points from image space to parameter space through parametric mapping. For line detection, the Hough transform employs polar coordinate representation (Equation 2):


(2) ρ=x·cos(θ)+y·sin(θ)

Among them:

*ρ* represents the vertical distance from the coordinate origin to the straight line.

*θ* represents the angle between the vertical line and the x-axis.

In this way, each point (x, y) in the image space is mapped to a sine curve in the Hough parameter space. The curves of collinear points in the parameter space will intersect at the same point (*ρ*, *θ*), which corresponds to a straight line in the image.

This study proposes a differentiable Hough transform-based loss function to enhance deep learning models’ linear feature extraction capability for roads. Traditional Hough transform, as a classical line detection algorithm, is widely adopted in computer vision and deep learning (Song et al., 2024). However, its discrete voting mechanism is inherently non-differentiable and incompatible with gradient-based optimization frameworks, necessitating adaptation.

To integrate the geometric prior of the Hough transform into our end-to-end deep learning framework, we address the core challenge of non-differentiability in the classical voting process. Our implementation details are as follows:

#### 3.2.1 Forward pass: standard Hough transform

Given a predicted probability map $P\in {\left[0,1\right]}^{\left(H\times W\right)}$ and a binary ground truth mask.

$G\in 0,1^{\left(H\times W\right)}$ , we first obtain a binary prediction mask M for the forward computation (Equation 3):


(3) $$M_{i,j}=I_{\left[P_{i,j}>0.5\right]}$$

Where I is the indicator function. This binarization step is non-differentiable.

Each active pixel (i,j) (where M_{i,j}=1 ) is mapped into the Hough parameter space. For line detection, we use the normal representation (Equation 4):


(4) ρ=i·cos(θ)+j·sin(θ)

discretize the parameter space: the angle θ is partitioned into $N_{\theta }$ bins (e.g., 180 bins from 0° to 180°), and the radius *ρ* is partitioned into $N_{\rho }$ bins with a resolution of Δρ (e.g., 1 pixel), up to a maximum value $\rho_{\max}=\sqrt{H^{2}+W^{2}}/2$ .

An accumulator matrix $\text{Accum}\in R^{N_{\theta }\times N_{\rho }}$ is constructed by casting votes. For each active pixel (i,j) and for each discrete angle $\theta_{k}$ , we compute the corresponding *ρ* value, determine its bin index l , and increment the accumulator (Equation 5):


(5) Accum[k,l]=Accum[k,l]+1

This process is performed for both the predicted mask M and the ground truth G, yielding $\text{Accu}m_{\text{pred}}$ and $\text{Accu}m_{\text{target}}$ .

The Hough loss $L_{\text{hough}}$ is then computed as the L1-norm difference between the two accumulator matrices, averaged over the batch (Equation 6):


(6) $$L_{\text{hough}}=\frac{1}{B\cdot N_{\theta }\cdot N_{\rho }}\sum_{b=1}^{B}\sum_{k=1}^{N_{\theta }}\sum_{l=1}^{N_{\rho }}|\begin{array}{l} \text{Accu}m_{\text{pred}}^{\left(b\right)}[k,l]\\ -\text{Accu}m_{\text{target}}^{\left(b\right)}[k,l] \end{array}|$$

Where B is the batch size.

#### 3.2.2 Backward pass: gradient approximation via STE

The critical step for differentiability lies in the backward pass. The gradient of the loss $L_{\text{hough}}$ with respect to the predicted probabilities P must be computed. The non-differentiable operation is the binarization $M=I_{\left[P>0.5\right]}$ .

We approximate the gradient using the Straight-Through Estimator (STE) (Huh et al., 2023). The STE defines a surrogate gradient for the thresholding function. Specifically, we treat the thresholding function as an identity function during backpropagation (Equation 7):


(7) $$\frac{\partial L}{\partial P}\approx \frac{\partial L}{\partial M}$$

The gradient $\frac{\partial L}{\partial M}$ can be derived by viewing the Hough transform as a linear voting operation. The chain rule is applied as (Equation 8):


(8) $$\frac{\partial L}{\partial M_{i,j}}=\sum_{k=1}^{N_{\theta }}\sum_{l=1}^{N_{\rho }}\frac{\partial L}{\partial \text{Accum}[k,l]}\cdot \frac{\partial \text{Accum}[k,l]}{\partial M_{i,j}}$$

Where $\frac{\partial L}{\partial \text{Accum}[k,l]}$ is simply the sign of the difference $\left(\text{Accu}m_{\text{pred}}[k,l]-\text{Accu}m_{\text{target}}[k,l]\right)$ from the L1 loss, and $\frac{\partial \text{Accum}[k,l]}{\partial M_{\left\{i,j\right]}}$ is 1 if the pixel (i,j) voted for bin (k,l) and 0 otherwise. In practice, this gradient is efficiently computed by a reverse Hough transform: the gradient $\frac{\partial L}{\partial \text{Accum}}$ is scattered back onto the image coordinates (i,j) along the same lines that were used in the forward vote.

#### 3.2.3 Computational overhead analysis

The introduction of the Hough loss adds non-negligible but manageable computational overhead. The complexity of the Hough transform is $O(N_{\text{active}}\cdot N_{\theta })$ , where $N_{\text{active}}$ is the number of active pixels. For a typical 1024×1024 image and $N_{\theta }=180$ , the forward and backward passes for the Hough loss introduce an approximately 20% increase in the per-epoch training time compared to a baseline using only pixel-wise losses. We deem this cost acceptable given the significant improvement in geometric accuracy and reduction in road discontinuities, as demonstrated in our results.

#### 3.2.4 Comparative cost vs. pixel-level losses

Unlike pixel-wise losses (e.g., BCE, L1) which operate at the native image resolution (H×W) , the Hough loss operates on a drastically down-sampled parameter space $\left(N_{\theta }\times N_{\rho }\right)$ . For example, with H=W=1024 and $N_{\theta }=180$ , $N_{\rho }\approx 725$ , the Hough space is over 8 times smaller than the image space. This makes the memory footprint of the Hough loss itself negligible. The primary cost is the voting procedure, which is highly parallelizable. The Hough loss is therefore computationally cheaper than many perceptual or style losses used in image generation tasks, while providing a strong, global geometric constraint that pixel losses inherently lack.

Unlike pixel-level losses such as L1 loss (Terven et al., 2025) and least squares loss (Wang et al., 2024), Hough transform loss operates in parameter space. This enables focus on linear structures with particular sensitivity to road-like features, effectively guiding models to learn geometric patterns. Simultaneously, it provides a global perspective that considers both local pixel accuracy and overall linear layout correctness. Through parameter space comparison, models generate more continuous and complete linear structures. Table 1 compares Hough transform loss advantages versus traditional losses for road extraction:

**Table 1:** Characteristics of different losses.

Name of loss	Pixel-level accuracy	Linear structure preservation	Robustness to noise	Global consistency
L1/L2 loss	High	Low	Low	Low
BCE LOSS	High	Low	Middle	Low
Dice loss	Middle	Middle	Middle	Middle
Hough loss	Middle	High	High	High
Adv loss	Low	Middle	High	High

**Figure 2:**
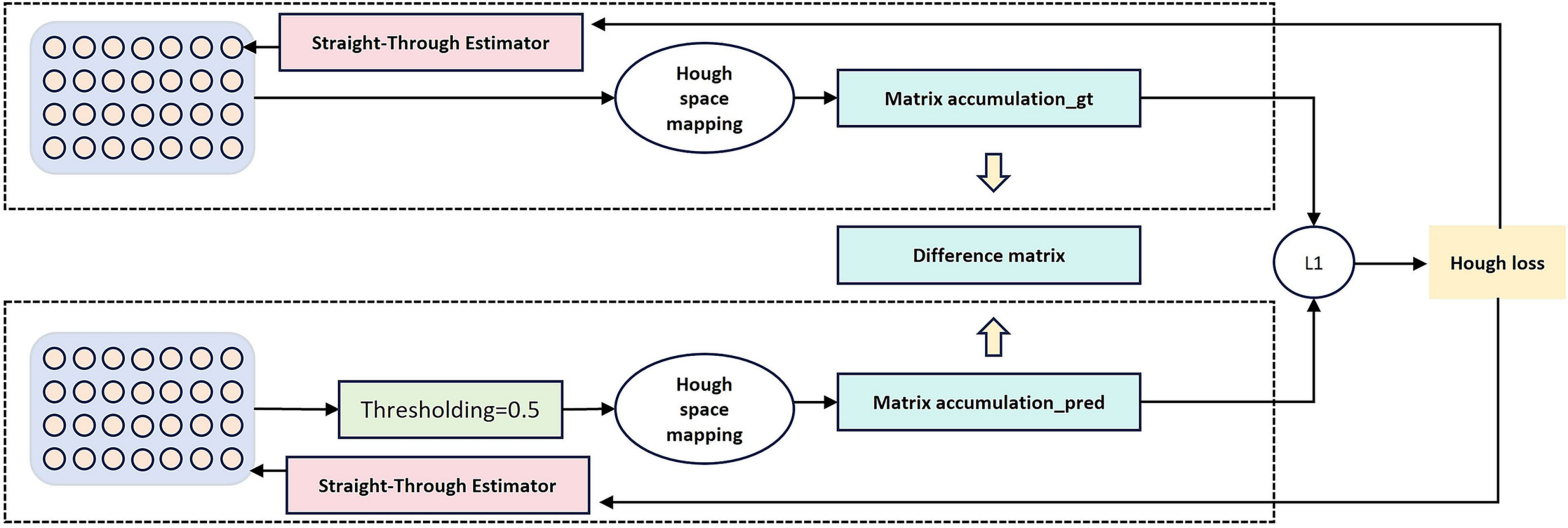
Illustration of the differentiable Hough-transform loss. Input features are projected into the Hough parameter space, where vote accumulation matrices are computed for both the ground truth mask and the predicted mask. The L1-norm difference between these matrices forms the Hough loss, which enforces linear and curvilinear structural consistency during training.

In our model, the Hough transform loss is combined with other loss functions, utilizing the complementary properties of different loss functions (Figure 2). Adversarial loss causes the generated road to appear realistic, serving as an implicit representation of the Hough transform in the discriminator network. This loss captures the geometric characteristics of the road through adversarial learning mechanisms. The discriminator network naturally develops sensitivity to linear features during the learning process, which is highly consistent with the line detection principle of the Hough transform. Specifically, when distinguishing between real roads and generated roads, the discriminator automatically learns to recognize typical geometric patterns of roads, such as straight lines, parallel lines, and regular intersections, which are precisely the features that the Hough transform focuses on.

### 3.3 Stage 1

**Figure 3:**
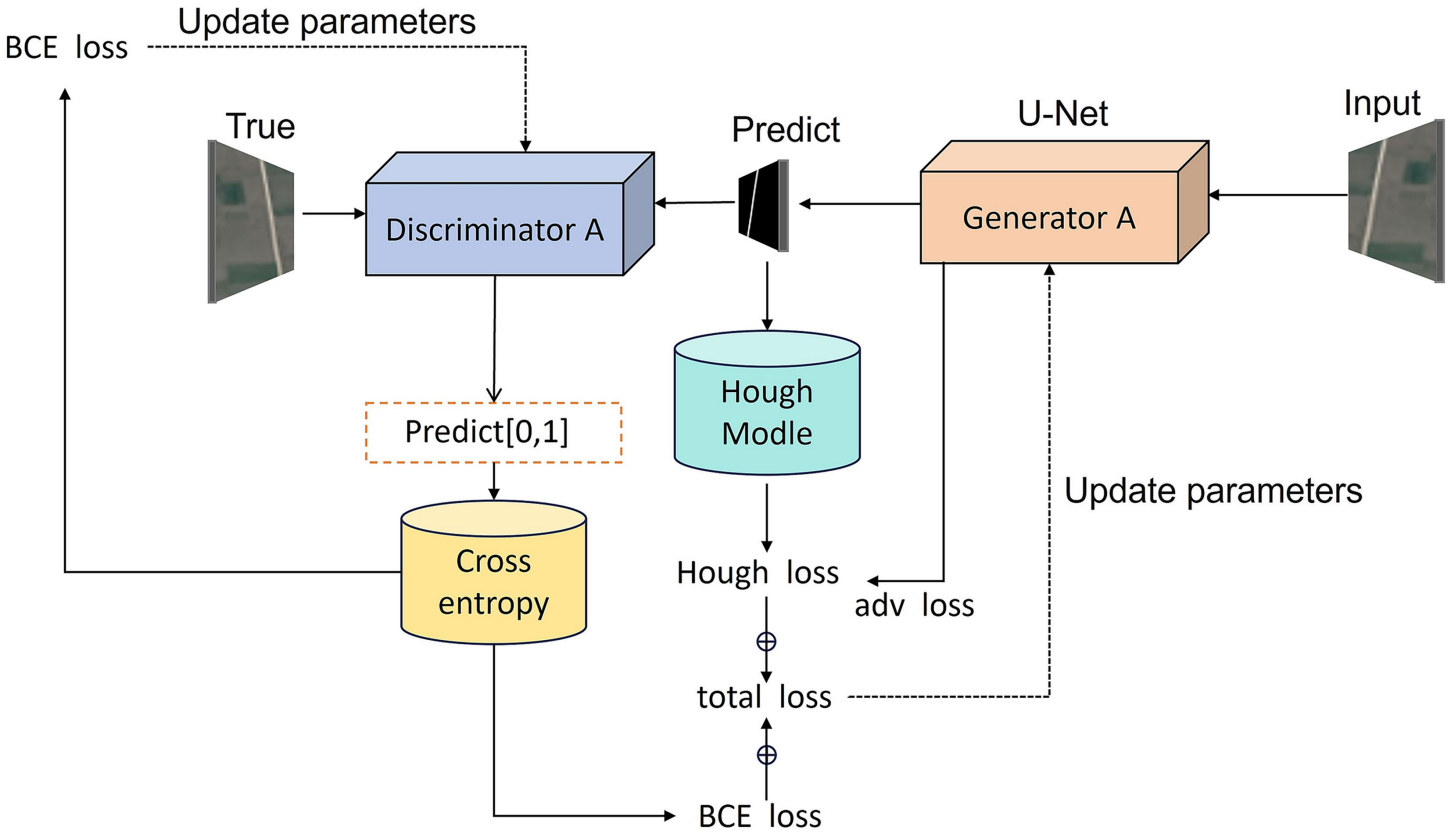
Workflow of the Stage 1 adversarial training process. The U-Net–based generator produces preliminary road masks from input satellite images, while the PatchGAN discriminator evaluates their realism against ground-truth masks. In parallel, the predicted outputs are passed through the Hough-transform module to calculate geometric loss. The total loss function combines adversarial, segmentation, Hough-transform, and geometric consistency terms, guiding the generator toward both pixel-level accuracy and global structural fidelity.

Our model enhances learning capability through dual neural network definition. In the Stage 1, we employ a standard U-Net generator trained against its discriminator, as shown in Figure 3. The U-Net architecture improves upon FCN (Fully Convolutional Network) (Long et al., 2015) and was initially applied to biological and medical image segmentation. It efficiently utilizes low-level image features to achieve accurate, rapid target extraction with limited training data.

The generator adopts the classic U-Net architecture comprising symmetrical encoder and decoder components:

Encoder section: Comprises 5 encoding blocks, each containing two 3 × 3 convolutional layers, batch normalization, and ReLU activation, followed by max pooling downsampling. Processing the input 3-channel satellite image, the encoder progressively extracts features while reducing spatial dimensions, with channel depths sequentially increasing to 64, 128, 256, 512, and 1,024.

Consists of 4 decoding blocks, each performing upsampling (via transposed convolution or bilinear interpolation) followed by two 3 × 3 convolutional layers. During decoding, skip connections concatenate same-level encoder features to preserve high-resolution spatial information. Channel depths decrease sequentially to 512, 256, 128, and 64.

Output layer: Produces a single-channel binary road mask through 1 × 1 convolution and Sigmoid activation.

The skip connections in U-Net directly transmit high-resolution features from the encoder to the decoder, effectively mitigating information loss in deep networks and preserving road boundary accuracy. The discriminator employs a PatchGAN architecture (Chen et al., 2023) to perform patch-level authenticity evaluation on the input image:

The discriminator receives satellite images and corresponding road masks (either real or generated) as inputs, initially concatenating them along the channel dimension. The network comprises 5 convolutional layers with channels of 64, 128, 256, and 512, respectively. It ultimately outputs a two-dimensional feature map representing the authenticity score for each patch. Finally, the Sigmoid activation function (Elfwing et al., 2018) maps these scores to the 0–1 range, indicating the discriminator’s confidence in the authenticity of each patch.

PatchGAN’s design concept segments an image into multiple overlapping patches for discrimination, rather than assigning a single true/false score to the entire image. This local discrimination mechanism enables the generator to focus more effectively on local road details and textures, thereby enhancing road boundary accuracy.

### 3.4 Stage 2

Inspired by DDU-Net (Wang et al., 2022), the second stage focuses on repairing discontinuities and suppressing isolated noise that remain after Stage 1. Unlike the first-stage GAN generator, this module is a simplified U-Net that receives the preliminary single-channel road mask from Stage 1 as input and outputs a refined mask of the same size. Rather than introducing new image-level features, it operates as a *secondary repairer*, concentrating on edge refinement, gap filling, and improving road connectivity.

To decouple the two stages, the output of Stage 1 is detached from the computation graph and directly fed into Stage 2, ensuring stable optimization without gradient interference. Training employs only supervised L1 loss against the ground-truth mask, deliberately excluding adversarial objectives to avoid unnecessary artifacts. In this cascaded design, Stage 1 emphasizes coarse segmentation with geometric constraints, while Stage 2 complements it by refining details and repairing local structures. Together, they form a dual-level optimization framework that significantly improves overall road continuity and integrity.

## 4 Data and training

### 4.1 Data source and preprocessing

**Figure 4:**
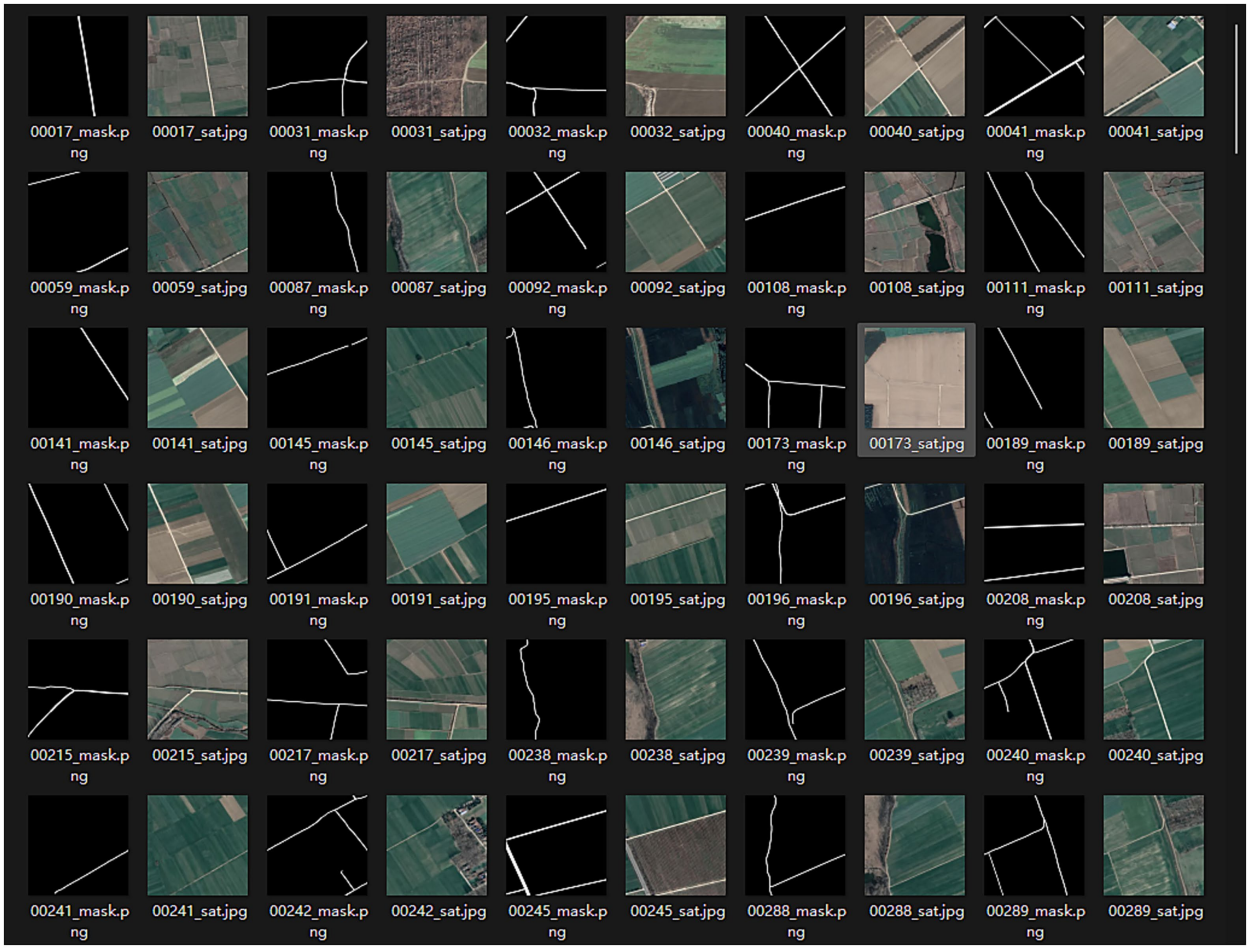
Preview of dataset images.

Due to the scarcity of rural roads in most current remote sensing datasets designed for cities, a specialized dataset, WHU RuR+ (Wang et al., 2025) is required for model training. WHU RuR + is a large-scale, high-resolution remote sensing dataset for rural road extraction. It contains 27,770 pairs of 1,024 × 1,024 satellite images with 0.3 m resolution and corresponding road annotations, covering 2620.71 km^2^ of rural areas in central China. A comprehensive analysis was conducted on the performance of state-of-the-art deep learning-based road extraction methods using the WHU RuR + dataset. Experimental results demonstrate that WHU RuR + presents significant challenges for large-scale rural road extraction. Furthermore, the dataset meets application requirements for rural road construction and exhibits substantial application potential. Sample data is shown in Figure 4.

**Figure 5:**
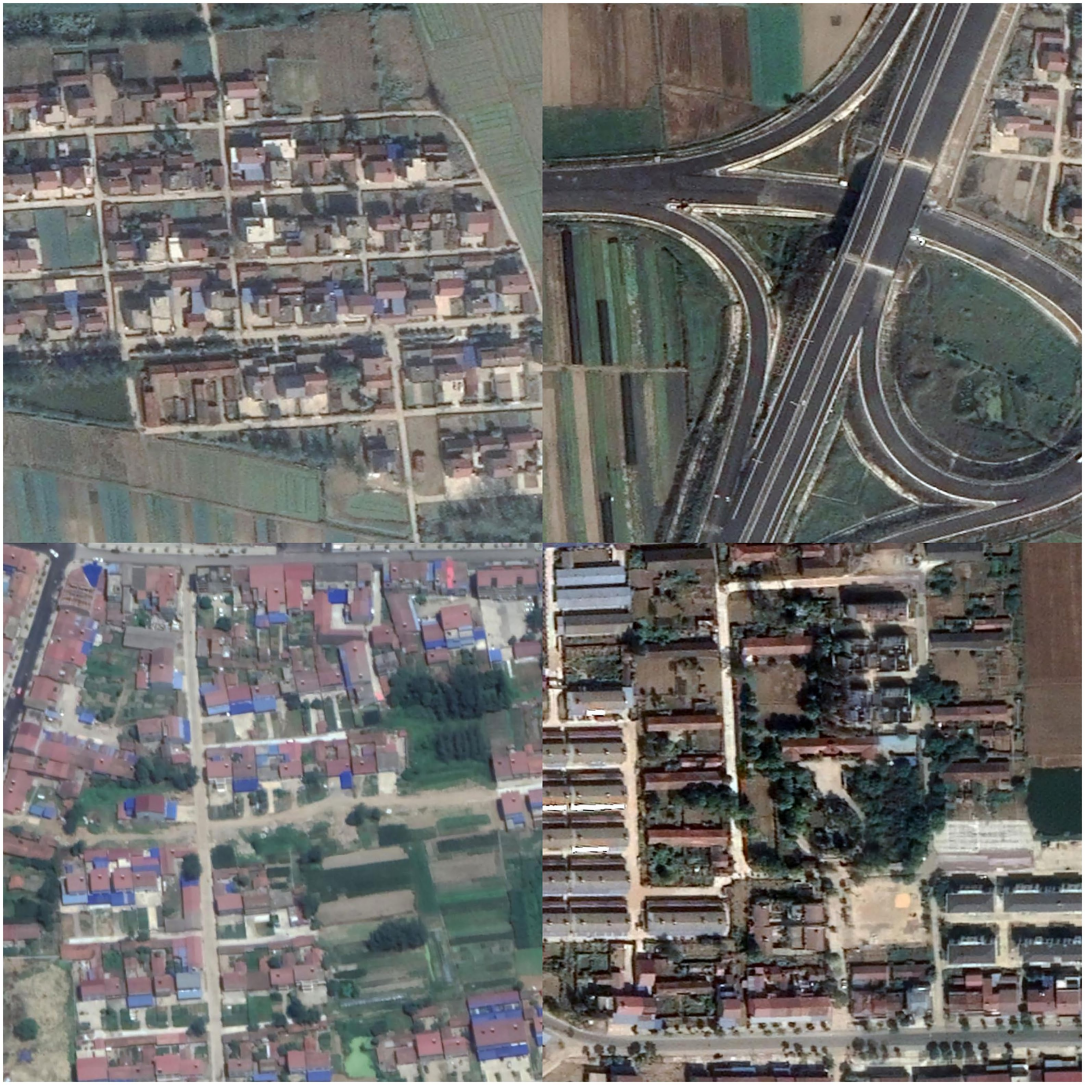
Data containing house noise.

To address the specific requirements and computational constraints of extracting roads in high-standard farmland, a targeted data selection strategy was applied to the WHU RuR + dataset. Although the dataset is large-scale (containing 27,770 images in total), a significant portion of the images include irrelevant objects such as houses, building complexes, and urban roads, as shown in Figure 5, which are not characteristic of farmland environments. Some images also suffer from severe occlusions or non-road interference, making them unsuitable for representing the road morphology and background features in “high-standard farmland” scenarios. Therefore, instead of using the entire dataset, we manually selected 120 images with typical farmland road characteristics, minimal occlusion, and high label quality for training, along with another 80 images for testing.

This strategy, while limiting the scale of the training data, was adopted based on the following considerations:

Task Specificity: Roads in high-standard farmland exhibit distinct geometric structures and background features. The selected subset is more representative of this application scenario.

Computational Efficiency: The dual-phase GAN combined with Hough transform loss involves high computational complexity. Large-scale training is impractical under limited computational resources (e.g., a single RTX 4090 GPU).

Noise Control: By excluding samples with significant non-farmland noise, the model can focus more effectively on the target features, thereby improving generalization in real farmland environments.

To mitigate overfitting caused by insufficient data during network training (Xiao et al., 2022), we applied a series of augmentation methods: RandomResizedCrop was used with a probability of 100% at each iteration, with a crop scale ranging from 0.8 to 1.0 to simulate multi-scale training; both RandomHorizontalFlip and RandomVerticalFlip were applied with the default PyTorch probability of 50% to improve directional invariance; RandomRotation was performed with 100% probability within a range of −30 to +30 degrees; and ColorJitter was applied with 100% probability, adjusting brightness, contrast, and saturation by ±20%, and hue by an offset of 0.1 radians, using bilinear interpolation to preserve color consistency. Finally, normalization was carried out using precomputed channel-wise means and standard deviations. Although the selected subset is relatively small, we mitigated the risk of overfitting through comprehensive data augmentation techniques—including random cropping, rotation, and color enhancement—which effectively increased the diversity of the training samples. Experimental results show that the model still achieves competitive performance on the test set, indicating that the quality and representativeness of the selected data compensate to some extent for the limited quantity. Nonetheless, we acknowledge that the generalization capability of the method under large-scale and highly complex environments requires further improvement. Future work will involve incorporating cross-regional data and introducing domain adaptation methods to enhance model robustness. We have open-source the specific code and dataset: https://github.com/badao162/UHGAN.

Although we excluded images with severe non-farmland structures (e.g., building complexes) to establish a baseline for typical farmland road extraction, we acknowledge that this limits the evaluation of model robustness in more general rural scenes. Our preliminary analysis indicates that performance degrades in such scenarios primarily because dense buildings often introduce severe occlusions, shadows, and complex intersections that break the continuity of road structures, challenging the model’s ability to infer global connectivity. Future work will explicitly test UHGAN’s robustness on diverse rural scenes containing non-farmland elements and explore architectural enhancements to better handle these complexities.

### 4.2 Parameter setting and training

The experiments were implemented in Python 3.8 with PyTorch on a NVIDIA GeForce RTX 4090 GPU. All input satellite images were resized to 1,024 × 1,024 pixels with three channels. A batch size of 1 was used to accommodate the high resolution of the data.

For the Stage 1 GAN, the generator was trained with the Adam optimizer (learning rate = 0.0013, betas = (0.5, 0.999)), while the discriminator used a smaller learning rate of 0.0003. For the Stage 2 refinement U-Net, the generator was optimized with Adam at a learning rate of 0.00013. To stabilize training, a cosine annealing learning rate scheduler ( $T_{\max}$ 120 epochs, $\mathrm{et}a_{\min}$ 0) was applied to all optimizers. The loss functions consisted of: Adversarial loss based on binary cross-entropy (BCE); segmentation loss; Hough transform loss; Geometric consistency loss. The training process lasted for 120 epochs, with early stopping when losses plateaued. Data loading was handled with the PyTorch DataLoader, using shuffled mini-batches.

To maintain road linearity and directional consistency, the Hough transform integrated with adversarial and segmentation losses constitutes part of the loss function in the model’s first stage. Predicted and ground truth masks are transformed into Hough parameter space, where their L1 distance is computed. This approach enhances preservation of road straight-line characteristics and directional coherence, focusing the model on road geometry rather than texture details. Additionally, geometric consistency loss improves invariance to geometric transformations, boosting road extraction robustness. The model applies 90° rotation to input images, processes them through the same generator, then inversely transforms the output. Minimizing the difference between original and inverse-transformed results ensures consistent road extraction across orientations. The Hough transform loss formula follows (Equation 9):


(9) $$\begin{array}{c} L_{\text{hough}}=\frac{1}{B\cdot \Theta \cdot R}\sum_{b=1}^{B}\sum_{\theta =1}^{\Theta }\;\sum_{r=1}^{R}|\text{Accu}m_{\text{pred}}^{\left(b\right)}(\theta ,r)\\ -\text{Accu}m_{\text{target}}^{\left(b\right)}(\theta ,r) \end{array}$$

Among them, B is the $\text{Accu}m_{\text{pred}}^{\left(b\right)}(\theta ,r)-\text{Accu}m_{\text{target}}^{\left(b\right)}(\theta ,r)$ accumulator of the predicted mask and the true mask in the Hough space, while B is the batch, the Θ number R of angle intervals, and the maximum radial distance.

The geometric consistency loss is as follows (Equation 10):


(10) $$L_{\mathrm{geo}}=\frac{1}{C\cdot H\cdot W}\sum_{c=1}^{C}\sum_{i=1}^{H}\sum_{j=1}^{W}|G{\left(x\right)}_{\left(c,i,j\right)}-T^{-1}(G(T(x){}_{\left(c,i,j\right)}))$$

Among them, G is the generator network, T is the geometric transformation, and $T^{-1}$ is the inverse transformation.

The total loss is as follows (Equation 11):


(11) $$L_{\text{total}}=\lambda_{1}\;\underset{\mathrm{adv}\;\text{loss}}{\underset{︸}{L_{\mathrm{adv}}}}+\lambda_{2}\;\underset{\mathrm{set}\;\text{loss}}{\underset{︸}{L_{\mathrm{seg}}}}+\lambda_{3}\;\underset{\text{Hough}\;\text{loss}}{\underset{︸}{L_{\text{hough}}}}+\lambda_{4}\;\underset{\mathrm{geo}\;\text{loss}}{\underset{︸}{L_{\mathrm{geo}}}}$$

Among them, there are weighting coefficients $\lambda_{n}$ (n = 1, 2, 3,4). Due to the limitation of training time, we adopted a small-scale grid search to determine the optimal hyperparameter configuration. Experimental results revealed that an excessively large weight for the geometric consistency loss actually led to a decline in model performance. Upon closer analysis, we attribute this to the fundamental differences between the optimization objectives guided by different loss functions.

The geometric consistency loss is designed to enhance the model’s robustness to geometric transformations (e.g., rotation and scaling), with its core constraint being that local pixels should remain consistent before and after transformation. However, the primary objective of the road generation task is to produce structurally coherent and well-connected road networks, which places greater emphasis on global topological correctness rather than strict pixel-level alignment. An overly large geometric consistency loss forces the generator to over-optimize local pixel alignment—for instance, enforcing unreliable correspondences in occluded or shadowed regions. This optimization direction diverges from high-level semantic goals, leading the model to generate overly conservative results that lack the ability to infer reasonable global road structures, thereby impairing connectivity and practical utility.

In contrast, the Hough loss explicitly incorporates prior knowledge of road geometry. By encouraging the model to generate responses aligned with line and curve features, it directly constrains the structural form of the output to better match the orientation and connectivity patterns of real roads. The adversarial loss guides the generator toward visually plausible global structures, while the segmentation loss provides pixel-level supervision. Within this framework, the geometric consistency loss should serve as an auxiliary component, with its weight kept relatively small to avoid constraining the model’s high-level semantic generation capability.

Therefore, we set $\lambda_{4}$ to a small value (0.001), which preserves a certain degree of geometric smoothness without undermining the model’s ability to capture global topological structures. The remaining loss weights—including the adversarial loss $\lambda_{1}$ , segmentation loss $\lambda_{2}$ , and Hough loss $\lambda_{3}$ —were tuned via grid search within the range {0.1, 0.5, 1}. This process yielded 27 experimental configurations in total, as illustrated in the Table 2. Finally, we selected $\lambda_{1}$ , $\lambda_{2}$ , and $\lambda_{3}$ as {0.1, 0.1, 1}.

**Table 2:** Grid parameter search results.

$\lambda_{1}$	$\lambda_{1}$ = 0.1			$\lambda_{1}$ = 0.5			$\lambda_{1}$ =1			$\lambda_{3},\lambda 2$
	$\lambda_{2}$ = 0.1	$\lambda_{2}$ = 0.5	$\lambda_{2}$ = 1	$\lambda_{2}$ = 0.1	$\lambda_{2}$ = 0.5	$\lambda_{2}$ = 1	$\lambda_{2}$ = 0.1	$\lambda_{2}$ = 0.5	$\lambda_{2}$ = 1
$\lambda_{3}$ = 0.1	0.756	0.751	0.747	0.749	0.754	0.743	0.752	0.748	0.732
$\lambda_{3}$ = 0.5	0.764	0.750	0.738	0.756	0.752	0.758	0.761	0.753	0.761
$\lambda_{3}$ = 1	0.747	0.754	0.775	0.745	0.747	0.772	0.749	0.752	0.767

The first row corresponds to the parameter selection for the adversarial loss, the second row represents the parameter selection for the segmentation loss, and the first column indicates the parameter selection for the Hough loss.

The second part of the model focuses on detail repair, employing pixel-level reconstruction loss to guide defect correction in the first-stage outputs. This stage directly compares pixel-level differences between the refined output and the ground truth mask. Using the L1 norm instead of the L2 norm reduces outlier influence, better preserves edge features, and prioritizes repairing broken road segments—optimizing connectivity and smoothness. Adversarial loss is deliberately excluded from this stage to prevent introduction of unnecessary high-frequency artifacts.

### 4.3 Evaluation metrics

To quantitatively evaluate the performance of the model, this paper employs Precision, Recall, and F1-Score as the core evaluation metrics. They are defined as follows Precision, Recall and F1-Score. These complementary metrics comprehensively assess the accuracy, completeness, and overall performance of the model in the farmland road extraction task. Detailed experimental results and analysis are presented in Section 5.

## 5 Conclusion

### 5.1 Result analysis

**Figure 6:**
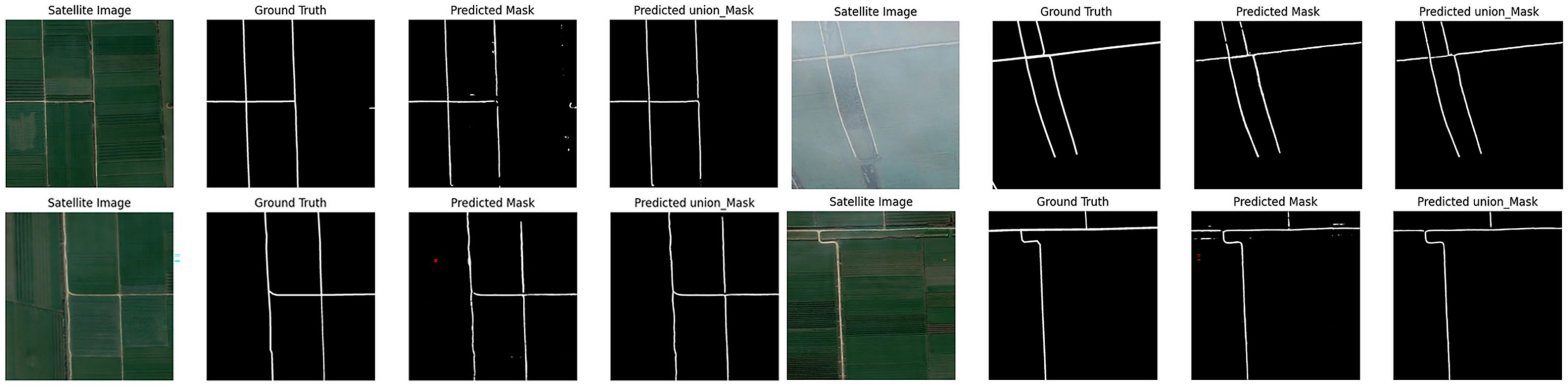
Model experiment results.

Using the constructed network to perform road extraction operations on the detection area, the results are shown in Figure 6.

**Figure 7:**
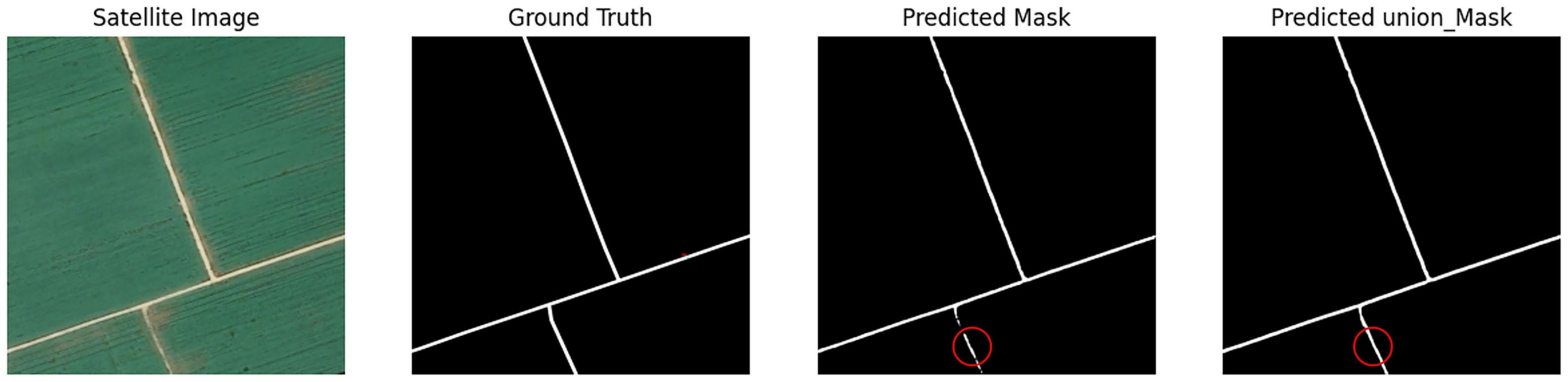
Poor model experimental results.

The first part of the joint learning model extracts accurate road geometries that align well with actual road contours. However, these extraction results contain numerous isolated points and discontinuities. The second model component specifically addresses these discontinuities and isolated points, achieving strong integrity without requiring additional repair algorithms. Both models maintain road trajectories consistent with actual roads, yielding smooth edges and reduced noise. Nevertheless, performance degrades noticeably in loose cultivated land areas. In these regions, road features lack sufficient visual clarity, are frequently occluded by vegetation, and often blend with surrounding field textures. Such conditions lead to weak feature representation in both the spectral and structural domains, which in turn reduces the model’s ability to distinguish roads from non-road areas. UHGAN is particularly sensitive to this problem because its adversarial component emphasizes the generation of visually plausible structures. When feature cues are ambiguous or suppressed, the generator tends to produce locally consistent textures at the expense of global structural correctness, resulting in missing or fragmented road segments. In other words, the model overfits to the dominant background patterns and fails to reconstruct the underlying road topology. After processing through the second model component, these discontinuities and edge noise exhibit effective restoration, as demonstrated in Figure 7.

**Figure 8:**
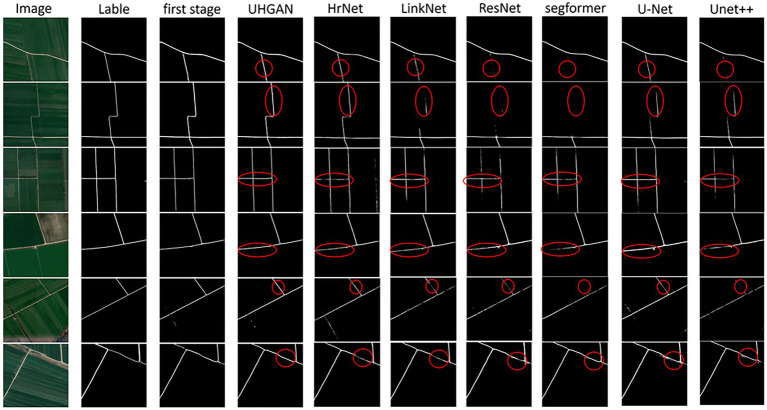
Comparison of road extraction results obtained by different methods. From left to right: input images, ground-truth labels, and the results of our proposed model (first 1 and Stage 1) as well as several baseline networks. In the Stage 1, the incorporation of adversarial learning and Hough transform loss enables the model to preserve the linear road structure and reduce omissions, although isolated points and discontinuities are still observed. In the second stage, the joint training strategy further alleviates these issues, leading to smoother boundaries, improved connectivity, and overall results that are more consistent with the ground truth compared to other competing approaches.

The comparative experimental results clearly demonstrate the performance differences among various methods in the task of road extraction. As shown in Figure 8, Our model demonstrates improved performance compared with baseline methods. In the Stage 1, by incorporating adversarial learning together with a Hough transform–based geometric constraint, the model learns to better capture the linear structural characteristics of roads. Compared with conventional segmentation networks (such as U-Net, UNet++, and Segformer), the Stage 1 results effectively reduce road omissions and successfully capture most of the main road segments. However, in some complex regions, a considerable number of isolated points and broken segments remain, leading to insufficient overall connectivity.

In the Stage 2, we adopt a joint training strategy that further refines the results while preserving the structural constraints introduced in the Stage 1. This strategy significantly alleviates the shortcomings of the Stage 1: isolated points and discontinuities are greatly reduced, road connectivity and integrity are enhanced, and the extracted boundaries appear smoother. Overall, the results are more consistent with the ground truth. Compared with other competing methods, our model exhibits stronger robustness and reliability in capturing fine road segments, maintaining structural continuity, and improving boundary accuracy, thereby providing a more realistic representation of road networks in remote sensing imagery.

### 5.2 Comparative experiment

To effectively evaluate extraction results, we employed precision, recall, and F1 score metrics on the preprocessed WHU RuR + dataset. Precision, Recall, and F1 Score constitute core classification model evaluation metrics, collectively quantifying identification accuracy and completeness for positive-class samples. Precision measures prediction exactness by calculating the proportion of true positives among predicted positives $\left(\frac{\mathrm{TP}}{\left[\mathrm{TP}+\mathrm{FP}\right]}\right)$ , preventing false alarms. Recall evaluates identification completeness through the proportion of actual positives correctly identified $\;(\frac{\mathrm{TP}}{\left[\mathrm{TP}+\mathrm{FN}\right]})$ , minimizing missed detections. The F1 score—harmonic mean of precision and recall—is calculated as $2\times \frac{\text{Precision}\times \text{Recall}}{\text{Precision}+\text{Recall}}$ . Ranging from 0 to 1, this balanced metric provides a comprehensive performance indicator particularly valuable for imbalanced data distributions.

These metrics exhibit complementary characteristics: excessively high precision may reduce recall (over-conservatism), while high recall may compromise precision (over-aggression). The F1 score optimizes the balance between them, achieving high values only when both precision and recall are strong. To benchmark our model’s superiority, we conducted systematic comparisons against established models including U-Net, ResNet (Takahashi et al., 2022), UNet++ (Zhou et al., 2018), SegFormer (Xie et al., 2021), LinkNet (Chaurasia and Culurciello, 2017), and HRNet (Sun et al., 2019). Experiments strictly followed identical train/validation/test splits, preprocessing procedures, and evaluation metrics (Precision, Recall, F1 Score). Results are presented in Table 3:

**Table 3:** Comparison experiment with other experiments.

Method	Precision	Recall	F1
UHGAN	0.826 ± 0.016	0.757 ± 0.013	0.789 ± 0.009
U-Net	0.759 ± 0.010	0.753 ± 0.012	0.756 ± 0.006
Resnet	0.798 ± 0.020	0.731 ± 0.031	0.762 ± 0.011
Unet++	0.771 ± 0.013	0.724 ± 0.019	0.747 ± 0.018
SegFormer	0.776 ± 0.023	0.744 ± 0.034	0.760 ± 0.010
LinkNet	0.804 ± 0.011	0.709 ± 0.011	0.754 ± 0.005
HRNet	0.813 ± 0.015	0.700 ± 0.021	0.752 ± 0.007

UHGAN achieves the highest average performance among the compared methods, with the highest F1-score (0.789 ± 0.009), strong precision (0.826 ± 0.016), and the best recall (0.757 ± 0.013), demonstrating that the dual-stage refinement strategy combined with Hough-transform–based global geometric constraints effectively enhances both road continuity and integrity. However, the analysis of confidence intervals reveals that UHGAN is not always the most stable model. For example, although its mean recall is the highest, the interval (±0.013) overlaps with that of U-Net (0.753 ± 0.012), and models such as LinkNet and HRNet exhibit narrower precision intervals, reflecting more consistent but less accurate predictions. This phenomenon arises because UHGAN emphasizes global geometric consistency rather than purely local pixel fitting; while this improves average structural accuracy, it also makes training more sensitive to dataset limitations and loss-weight tuning, resulting in slightly higher variance. In summary, UHGAN provides the strongest overall balance between precision and recall, but its superior mean performance comes at the cost of reduced stability in certain cases—a limitation that can be mitigated in future work through larger-scale training (Shen et al., 2024) and refined optimization strategies (Zheng et al., 2025).

To verify the effectiveness of each component in the two-stage joint training framework (UHGAN) proposed in this paper, we designed a systematic ablation experiment (the results are shown in Table 4).

**Table 4:** Ablation experimental results.

Remaining modules	Precision	Recall	F1
UHGAN	0.826 ± 0.016	0.757 ± 0.013	0.789 ± 0.009
Union-GAN without Stage 2 refinement	0.828 ± 0.023	0.732 ± 0.017	0.777 ± 0.012
GAN without Assembling loss (Assembling loss = $\lambda_{1}\;L_{\mathrm{adv}}+\lambda_{2}\;L_{\mathrm{set}}+\lambda_{3}\;L_{\text{hough}}+\lambda_{4}\;L_{\mathrm{geo}}$ )	0.801 ± 0.016	0.757 ± 0.015	0.778 ± 0.010
GAN (no refinement, no Assembling loss)	0.821 ± 0.026	0.680 ± 0.021	0.743 ± 0.020

The Assembling loss is composed of adversarial loss, segmentation loss, Hough transform loss, and geometric consistency loss.

Our full model, UHGAN, which combines a Stage 1 GAN with Hough-transform Assembling loss (Assembling loss = $\lambda_{1}\;L_{\mathrm{adv}}+\lambda_{2}\;L_{\mathrm{set}}+\lambda_{3}\;L_{\text{hough}}+\lambda_{4}\;L_{\mathrm{geo}}$ ) and a Stage 2 refinement U-Net, achieves the best overall balance, with a precision of 0.826, recall of 0.757, and F1-score of 0.789. Removing the Stage 2 refinement (Union-GAN without Stage 2 refinement) leads to a drop in recall to 0.732 and a slight decrease in F1-score to 0.777, highlighting the importance of the second-stage module in maintaining road continuity and eliminating isolated fragments. When the Hough-transform Assembling loss is removed while retaining the Stage 2 refinement [GAN without Assembling loss (Assembling loss = $\lambda_{1}\;L_{\mathrm{adv}}+\lambda_{2}\;L_{\mathrm{set}}+\lambda_{3}\;L_{\text{hough}}+\lambda_{4}\;L_{\mathrm{geo}}$ )], the model achieves a precision of 0.801 ± 0.016 and a recall of 0.757 ± 0.015, resulting in an F1-score of 0.778 ± 0.010. Compared with UHGAN, the slightly lower F1-score indicates that the Assembling loss plays a critical role in capturing global structural information and maintaining balanced performance, preventing the model from overfitting to local features. The simplest baseline, which omits both the refinement stage and the Assembling loss (GAN (no refinement, no Assembling loss)), shows the most severe precision–recall imbalance, with recall dropping to 0.680 and F1-score sharply decreasing to 0.743, confirming that neither component alone is sufficient for robust performance. Overall, these ablation results demonstrate the complementary effects of the two design choices: the Stage 1 GAN with Assembling loss enforces global geometric consistency, while the Stage 2 refinement U-Net repairs discontinuities and suppresses noise, together enabling coherent and continuous road extraction with superior structural integrity (Equation 12).


(12) $$\begin{array}{c} \text{Assembling loss}=\lambda_{1}\;L_{\mathrm{adv}}+\lambda_{2}\;L_{\mathrm{set}}+\lambda_{3}\;L_{\text{hough}}\\ +\lambda_{4}\;L_{\mathrm{geo}}(\lambda_{1},\lambda_{2},\lambda_{3},\lambda_{4}=0.1,1,1,0.001) \end{array}$$

### 5.3 Future work

Although the UHGAN model shows competitive results in high-standard farmland road extraction—particularly in maintaining road geometric continuity and suppressing noise—several limitations remain, leaving room for future exploration. First, constrained by computational resources and data availability, this study was conducted on a relatively small manually curated subset of 200 images. While data augmentation was employed to mitigate overfitting risks, the model’s generalization ability in larger-scale and more complex farmland scenarios still requires further validation. Second, the model continues to struggle with road segments subject to severe occlusion or those exhibiting strong similarity to background textures. In particular, in areas with loose soil or dense vegetation coverage, the model’s perceptual and reasoning capabilities remain inadequate.

Future research will focus on the following directions: 1. constructing larger-scale, multi-regional, and multi-temporal farmland road datasets (Shen et al., 2025), combined with domain adaptation techniques (Zhang et al., 2022) to enhance robustness under varying geographic environments and imaging conditions; 2. exploring Transformer-based architectures (Wang et al., 2024) to replace or augment the current U-Net backbone, thereby improving the modeling of long-range dependencies and global contextual information (Chen et al., 2023); 3. further refining the differentiable implementation of the Hough loss to better accommodate curved roads and complex topological structures; 4. advancing toward real-time applications, such as integration into agricultural drones (Askarzadeh et al., 2025) or mobile terminal systems (Yu et al., 2023), to provide instant and high-precision road information in support of precision agriculture and rural planning.

## Data Availability

The datasets presented in this study can be found in online repositories. The names of the repository/repositories and accession number(s) can be found at: https://www.scidb.cn/en/detail?dataSetId=9824c6ce552244e087dd5d4f7ad23883.
